# Evaluating the informatics for integrating biology and the bedside system for clinical research

**DOI:** 10.1186/1471-2288-9-70

**Published:** 2009-10-28

**Authors:** Vikrant G Deshmukh, Stéphane M Meystre, Joyce A Mitchell

**Affiliations:** 1 Department of Biomedical Informatics, University of Utah, 26 South 2000 East, HSEB Room 5775, Salt Lake City, UT 84112, USA; 2 Health Sciences Information Technology Services, University of Utah, 585 Komas Drive Suite 204, Salt Lake City, UT 84108, USA

## Abstract

**Background:**

Selecting patient cohorts is a critical, iterative, and often time-consuming aspect of studies involving human subjects; informatics tools for helping streamline the process have been identified as important infrastructure components for enabling clinical and translational research. We describe the evaluation of a free and open source cohort selection tool from the Informatics for Integrating Biology and the Bedside (i2b2) group: the i2b2 hive.

**Methods:**

Our evaluation included the usability and functionality of the i2b2 hive using several real world examples of research data requests received electronically at the University of Utah Health Sciences Center between 2006 - 2008. The hive server component and the visual query tool application were evaluated for their suitability as a cohort selection tool on the basis of the types of data elements requested, as well as the effort required to fulfill each research data request using the i2b2 hive alone.

**Results:**

We found the i2b2 hive to be suitable for obtaining estimates of cohort sizes and generating research cohorts based on simple inclusion/exclusion criteria, which consisted of about 44% of the clinical research data requests sampled at our institution. Data requests that relied on post-coordinated clinical concepts, aggregate values of clinical findings, or temporal conditions in their inclusion/exclusion criteria could not be fulfilled using the i2b2 hive alone, and required one or more intermediate data steps in the form of pre- or post-processing, modifications to the hive metadata, etc.

**Conclusion:**

The i2b2 hive was found to be a useful cohort-selection tool for fulfilling common types of requests for research data, and especially in the estimation of initial cohort sizes. For another institution that might want to use the i2b2 hive for clinical research, we recommend that the institution would need to have structured, coded clinical data and metadata available that can be transformed to fit the logical data models of the i2b2 hive, strategies for extracting relevant clinical data from source systems, and the ability to perform substantial pre- and post-processing of these data.

## Background

With an increased emphasis on translating the discoveries from basic sciences into clinical applications [1,2], there is a need for informatics tools that enable the efficient exchange of data, information and knowledge between different clinical and research entities in an organization, as well as, between different organizations [3,4]. Estimating cohort sizes and selecting appropriate cohorts of patients/subjects are important tasks in clinical research; several commercial and open source software tools are available for this purpose. The present work describes the implementation and evaluation of one such open source tool at the University of Utah Health Sciences Center (UUHSC).

Informatics for Integrating Biology and the Bedside (i2b2) [5] is one of the seven National Centers for Biomedical Computing funded by the National Institutes of Health, and is based at the Brigham and Women's Hospital (Boston, MA) [6]. The i2b2 center focuses on developing a new informatics framework to bridge clinical research data with basic sciences research data, and on driving biology projects that serve as test beds for the framework. One of the most visible components of the i2b2 informatics framework is an open source patient cohort selection tool called the i2b2 workbench, which is a modular, user-friendly tool that allows graphical querying and visualizing of clinical data. The complete system consists of a modular server component called the i2b2 'hive,' and of a cross-platform Java [7] client called the i2b2 workbench. The latest version of the i2b2 hive (1.3) also includes a web client that is functionally similar to the Java client.

The i2b2 hive consists of interoperable 'cells' communicating with one another in a Service-Oriented Architecture [8,9] (SOA), and of a persistent data staging area called the Clinical Research Chart (CRC). An Oracle database (Oracle Corp., Redwood Shores, CA) or a Microsoft SQL Server database (Microsoft Corp., Redmond, WA) serves as a repository for data from a variety of different information systems used in clinical operations and research. Each i2b2 cell is the basic building block of the hive, and encapsulates business logic and access to data behind web services standards such as Representational State Transfer [10] (REST), or Simple Object Access Protocol [11] (SOAP). Many cells within the hive have corresponding client-side components, which the end-users can interact with in the i2b2 workbench. The architecture and design of the i2b2 hive were inspired by the Research Patient Data Registry [12] (RPDR), a visual query tool [13,14] developed at Partners Healthcare.

In order to investigate the generalizability of the i2b2 model, it was important to study whether it could be successfully implemented at an institution not involved in its development. The Department of Biomedical Informatics at the University of Utah, one of the leading informatics centers in the U.S. [15,16], was selected for its significant informatics expertise, the availability of an existing clinical data repository and for its well-developed resources in both the clinical and research domains. A key resource at the University of Utah Healthcare System is the Enterprise Data Warehouse (EDW), which integrates data from over 200 disparate clinical, financial and ancillary systems. Two of the major Electronic Medical Record (EMR) systems contributing data to the EDW include Cerner Millennium (Cerner Corp., Kansas City, MO) in the inpatient setting and Epic (Epic Systems Corp., Verona, WI) in the ambulatory setting. In addition to these two major EMR systems, there are several legacy systems which have been integrated within the EDW over a period of time. The EDW contains records for over 2 million patients that were extracted from various EMR and ancillary systems since 1995. The partnership between i2b2 and the Department of Biomedical Informatics involved installing and evaluating version 1.2 of the i2b2 hive at the UUHSC, and we have since upgraded to version 1.3. The evaluation described in the present work included the time and effort needed to install and adapt the i2b2 hive, a comparison of the data models and terminology systems of existing databases at the University of Utah with those found in the i2b2 hive, and the suitability of the i2b2 hive and workbench for fulfilling real clinical data requests from researchers as use cases.

## Methods

### Hive setup, development and staging environments

**Figure 1:**
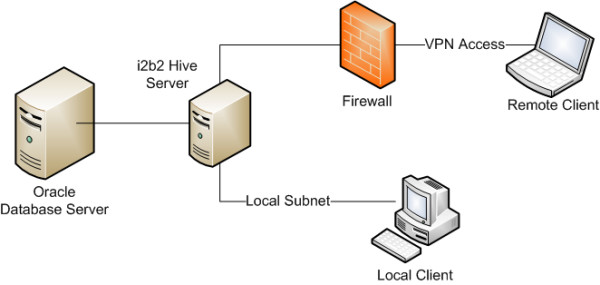
**Hive topology**. Hive topology of the staging environment showing a local client and a remote client connected to the i2b2 hive, with the database running on a separate node.

The i2b2 hive architecture [6] is highly flexible and configurable, and allows for various components of the hive to be set up under different topologies as long as the core components are able to communicate with one another. The suggested i2b2 hive setup consists of a single server hosting Apache Tomcat [17] and JBoss Application Server [18] with Apache Axis2 [19] & GridSphere Portal Framework [20], and Oracle Express Edition (XE) [21], running on Linux [22] or Microsoft Windows (Microsoft Corp., Redmond, WA). In order to create a development environment, the i2b2 hive was installed in the suggested configuration on a Linux workstation. For the staging environment, a slightly different configuration was adopted with the hive applications and the database running on separate servers (Figure 1). The staging environment consisted of the i2b2 hive software installed on a Red Hat Enterprise Linux 4 platform [23] hosted on a quad AMD Opteron server [24] with 8 GB of RAM, gigabit Ethernet connectivity and dedicated storage. This server was also setup with Hewlett-Packard OpenView monitoring system [25] for proactive monitoring, with alerts and pages sent to the EDW team. Documentation of the server setup was done by following standard IT practices. The development database was used for prototyping all the SQL queries and procedures used in populating the main i2b2 repository (called the Clinical Research Chart) as well as for testing any additions/modifications to the i2b2 metadata.

### De-identification and access to data

Although the study was approved by the Institutional Review Board (IRB) to include identifiable patient information, clinical data were partially de-identified before adding them to the i2b2 hive, in order to reduce risk. De-identification was accomplished by using internal system-generated IDs in the place of actual medical record numbers and hospital visit numbers. The dates of birth were rounded to the year, and names of patients, providers, and specific locations in the hospital were excluded from the dataset used to populate the hive. The actual dates and times on all other events recorded in the fact table of the i2b2 repository were left intact, since these attributes were necessary in order to recreate the sequence of recorded events and for generating the timeline in the i2b2 workbench application. The measures used in de-identification can reduce risk, but in order to fully meet the date criteria of the HIPAA Safe Harbor method, the date/time of each observation would also have to be obscured or obfuscated by another method, and such level of de-identification was not performed in the present investigation.

Access to the i2b2 hive data was limited to the principal investigator and co-investigators by creating accounts with appropriate access privileges through GridSphere. The servers hosting the i2b2 hive and the Oracle database were setup behind a firewall, and computers used to access the i2b2 hive had to be either physically connected on the same network subnet protected from external access by a corporate firewall, or connected to this subnet over a Virtual Private Network (VPN) connection with strong encryption (Figure 1).

### Evaluation based on requests for research data

In addition to architecting and supporting the software infrastructure for providing data for research and operations, the EDW team works directly with researchers in preparing datasets used in research. Requests for clinical data are received by the EDW in an electronic form through a web-based data request tool, or as appropriate IRB forms filled out on paper directly from researchers, and all requests are then reviewed by the IRB. A total of 337 requests for clinical data received electronically through the web-based data request tool during the period 2006-2008 were considered for this evaluation. Data-requests that included operational or financial components (e.g. 'top ten referring facilities by patient volume & diagnoses') or those marked for delivery through the institution's 'enterprise-wide reporting infrastructure,' (e.g. automated daily/weekly reports) for non-research purposes were excluded, since these were beyond the scope of the present investigation. Following the screening process, 27 research-related data requests (listed in Table 1, descriptions of individual data requests can be found in additional file 1) were used to evaluate the i2b2 hive on the basis of the following criteria:

**Table 1:** Types of data requests in the sample (descriptions of individual data requests can be found in 'additional file 1').

Request #	Patient counts	Actual data	Diagnoses	Procedures	Medications	Labs	Demographics
1	**•**	**•**			**•**		**•**
2	**•**		**•**				
3	**•**	**•**	**•**				**•**
4	**•**			**•**	**•**		
5	**•**	**•**		**•**	**•**		
6	**•**	**•**	**•**				**•**
7	**•**	**•**		**•**		**•**	
8	**•**	**•**		**•**		**•**	**•**
9	**•**	**•**		**•**		**•**	**•**
10	**•**	**•**	**•**		**•**		**•**
11	**•**	**•**	**•**	**•**		**•**	**•**
12	**•**	**•**		**•**		**•**	**•**
13	**•**	**•**	**•**	**•**		**•**	**•**
14	**•**	**•**	**•**		**•**		**•**
15	**•**	**•**	**•**		**•**	**•**	**•**
16	**•**	**•**	**•**	**•**			**•**
17	**•**		**•**	**•**			
18	**•**	**•**					**•**
19	**•**	**•**	**•**	**•**			**•**
20	**•**		**•**				
21	**•**		**•**	**•**			
22	**•**		**•**				**•**
23	**•**		**•**				**•**
24	**•**			**•**			
25	**•**	**•**			**•**		**•**
26	**•**			**•**			
27	**•**	**•**		**•**			**•**

**Total**	**27**	**18**	**15**	**15**	**7**	**7**	**18**

**%**	**100%**	**66.6%**	**55.5%**	**55.5%**	**25.9%**	**25.9%**	**66.6%**

- whether the PI had requested counts or actual clinical data, or both;

- the types of clinical data elements that were requested (diagnoses, procedures, medications and/or laboratory examinations);

- whether the request included institution-specific criteria, relying on specific data sources which may not be found elsewhere;

- features of the query based on a given data request (temporal criteria, exclusion criteria, calculated fields, criteria based on aggregate data);

- whether the entire data request could be performed using the i2b2 alone without significant additions/modifications;

- and, reasons when it was not possible to complete the entire data request using i2b2 alone (additional data attributes required, modifications of the i2b2 hive metadata required, additional pre-processing required, and/or additional post-processing required).

The diagnoses, medications, laboratory examinations and patient demographics needed in the study were extracted from the EDW, transformed to fit the i2b2 hive data model, and then loaded into the i2b2 Clinical Research Chart in the development database. The content in the development database was then validated against source data, and after successful validation, loaded into the database in the staging environment. The transformation process involved mapping data fields from the source systems, to the minimal set of attributes used in the i2b2 star schema model for the fact table (observation_fact) and the various dimension tables.

## Results

### Evaluation based on research data requests

Upon analyzing the 27 selected research data requests (Table 1), it was observed that about 44% (12) of these data requests could be completed using the i2b2 workbench/hive alone, without requiring significant additions/modifications to the hive metadata (i.e. ontology), as mentioned in Table 2. About 67% (18) of the data requests needed actual patient data in addition to the counts, whereas the rest had only requested counts. The most frequently requested data elements were demographics [67% (18)] diagnoses [56% (15)], procedures [56% (15)], medications [26% (7)] and laboratory tests [26% (7)]. The default installation of the i2b2 hive contains metadata for all of the above types of data elements, and was adequate for successfully completing many of the data requests in the sample, without requiring extensive modifications to these metadata.

**Table 2:** Data requests that could be performed with the i2b2 workbench or that required significant modifications.

Request #	No significant modifications	Modifications required							
		
		Institution specific	Pre-processing	Post-processing	Exception conditions	Temporal conditions	Calculated fields	Additional attributes	Metadata modifications
1			**•**	**•**				**•**	**•**
2	**•**					**•**			
3			**•**	**•**			**•**	**•**	**•**
4				**•**		**•**			
5	**•**				**•**				
6	**•**								
7			**•**	**•**			**•**		**•**
8			**•**	**•**		**•**	**•**		**•**
9	**•**			**•**					
10	**•**			**•**					
11			**•**	**•**		**•**	**•**	**•**	**•**
12			**•**					**•**	**•**
13		**•**	**•**	**•**		**•**	**•**	**•**	**•**
14		**•**	**•**	**•**				**•**	**•**
15	**•**								
16	**•**								
17	**•**								
18			**•**				**•**	**•**	**•**
19	**•**					**•**			
20		**•**	**•**			**•**		**•**	**•**
21		**•**	**•**					**•**	**•**
22		**•**	**•**	**•**	**•**	**•**	**•**	**•**	**•**
23		**•**	**•**	**•**	**•**	**•**		**•**	**•**
24	**•**					**•**			
25		**•**	**•**	**•**		**•**	**•**	**•**	**•**
26	**•**			**•**		**•**	**•**		
27	**•**			**•**		**•**			

**Total**	**12**	**7**	**14**	**15**	**3**	**13**	**9**	**12**	**14**

**%**	**44.4%**	**25.9%**	**51.8%**	**55.5%**	**11.1%**	**48.1%**	**33.3%**	**44.4%**	**51.8%**

Among the data requests that could not be fulfilled with the i2b2 workbench without significant modifications (Table 2), 26% (7) had one or more institution-specific criteria (e.g. data from the Utah Population Database, specific hospital clinics or service lines, etc.), and 48% (13) had one or more temporal criteria that could not be faithfully reproduced using the graphical interface of the i2b2 workbench alone. Such query criteria often required pre-processing and/or post-processing of data in order to answer the research questions. An example of pre-processing can be found in data request #1 where the inclusion criterion of '*IV *antibiotics' required a pre-coordination of two separate structured data elements, the Multum drug codes for antibiotics and the routes of administration. An example of post-processing can be found in data request #4, where the data set consisted of patients who had received Rifampicin *after *their hip or knee surgery. In order to fulfill this particular data request using i2b2, one would have to initially select patients based on their surgical procedure and the administration of Rifampicin, followed by a further sub-selection of the population by grouping the resulting data set by their 'visit' and then selecting only the ones who had received Rifampicin *after *their surgical procedure. While the timeline view in i2b2 would allow the user to manually select individual patients in whom the drug was administered after surgery, the graphical interface itself imposes limitations that prevent all query conditions from being satisfied without post-processing in the form of extensive manual intervention, which may not be practical when selecting large research cohorts.

During the analysis of the sample, it was also discovered that 52% (14) of the data requests required some type of pre-processing of the data, 56% (15) of the requests required some post-processing, and 33% (9) of the requests required both pre- and post-processing in order to fulfill the data request. Another 33% (9) of the requests included calculated fields, and 44% (12) of the requests required additional attributes in order to accommodate one or more complex inclusion criteria. An example of calculated fields can be found in data request #3 where the PI was interested in obtaining a set containing outliers (defined as 3 standard deviations for the length of stay) and another set excluding outliers, which would require first extracting data using their initial inclusion criteria, calculating the standard deviation and then excluding the outliers from one data set based on this calculation. In addition to pre- and post-processing steps, 52% (14) of the requests required one or more additions to the i2b2 metadata.

## Discussion

### Selection of patient cohorts

One of the greatest strengths of the i2b2 hive is the ability to provide estimates of cohort sizes by making changes to the inclusion/exclusion criteria, which is an important part of the pre-research process. From Table 1, cases where the researchers had only requested counts of patients accounted for 33% (9) of all the research data requests in the sample--typically investigations in a pre-research stage--while the other 67% (18) needed counts as well as actual data sets. Even in data requests where researchers request actual patient data in addition to counts, it is common for researchers to go through several iterations of a dataset, since initial analysis may sometimes lead to a revision of one or more inclusion or exclusion criteria. In both types of data requests, iterations can be time-consuming for both the researchers and the data analysts who obtain these data. The true benefit of a tool like the i2b2 hive can be realized by substantially reducing the overall time between the initial estimates of cohort sizes and the availability of the final data sets used in clinical research.

From Table 2, a large number of data requests (2, 5, 6, 9, 10, 15, 16, 17, 19, 24, 26 and 27) could be performed with the hive without requiring any additions to the i2b2 ontology. These requests represented 44% (12) of the total number of research data requests in the sample, and could allow offloading a substantial amount of effort from the hands of data analysts who typically perform such tasks, and represent one of the biggest advantages of using a graphical cohort selection tool such as the i2b2 workbench.

### Enhancements to the metadata/terminologies

The Ontology Management cell in the i2b2 hive is designed to accommodate controlled medical terminologies, local vocabularies, as well as user created terminologies. The i2b2 hive is made available with sample metadata containing medical vocabularies for diagnoses and procedures (International Classification of Diseases, Ninth Revision, Clinical Modifications (ICD-9-CM) [26]), medications (Cerner Multum [27] and National Drug Code (NDC) [28]), and laboratory tests (Logical Observations Identifiers Names and Codes (LOINC) [29]). In addition, terminologies for patient demographics, including age, gender, language, marital status, race, religion, vital status and ZIP codes are also provided with the i2b2 hive. While the included terminologies may suffice when selecting patient cohorts based on simple criteria involving diagnoses and a limited number of demographic data elements, additional local/custom terminologies are needed to fully use the capabilities of the i2b2 software and allow querying institutional data extracted from various administrative and clinical information systems, which use such terminologies.

About 52% (14) of the data requests used in this evaluation required some form of enhancement to the metadata before the underlying data could be used within the hive. Many of the terminology-related challenges encountered were related to the inconsistent use of controlled medical vocabularies when representing laboratory tests and results in the different clinical information systems. In particular, legacy systems that included local terminologies with inadequate mappings to controlled medical terminologies were more difficult to integrate within the i2b2 hive. Similarly, in regard to medications, the use of the Multum hierarchy for therapeutic classes and drug names, combined with NDC codes was suitable for integrating data from our inpatient system, which uses the same terminologies, but was problematic when trying to integrate data from the ambulatory care system--Epic--which uses Wolters Kluwer MediSpan [30] as it's underlying terminology. Integrating laboratory data posed similar problems, since the i2b2 hive uses LOINC as the underlying terminology, but vocabularies from legacy systems that contribute laboratory data to the data warehouse do not completely map to LOINC codes.

An additional challenge in adding local terminologies to the hive was to recreate them in a manner that would allow easy navigation in the ontology navigation plug-in of the i2b2 workbench. With legacy local terminologies, there were often no distinct hierarchies, or the hierarchies were organized in ways less familiar to clinicians and researchers. Some of the legacy systems contained coding approaches that had been modeled on the basis of financial transactions rather than clinical care--a vestigial consequence of the evolution of EMR systems from specialized hospital financial systems that preceded them--or the descriptions for some of the codes were not informative enough, and as such, could not be included directly into the hive metadata without standardizing their nomenclature, and/or mapping to controlled medical terminologies for these terms. Therefore, the i2b2 software implementation of terminologies allows for great flexibility, and some of the challenges in efficiently using metadata in i2b2, and related to data integration and metadata management in general.

### Temporal criteria in queries

Temporal criteria such as ranges of dates were relatively easy to model in the queries using the i2b2 workbench, and 48% (13) of the data requests in the sample contained temporal criteria. Data requests that included more complex temporal conditions in the inclusion/exclusion criteria required some amount of pre-processing and/or post-processing. In data request #25, for example, the researcher had requested data from the first week of every month for a period of one year, which would require multiple query conditions to be included, including dynamically determining the first week of a given month. Such logic can be expressed in a single SQL query, but would require multiple runs using the i2b2 workbench to get separate datasets, which would then have to be combined together. In another example, the events in the inclusion and exclusion criteria had to have occurred in a certain sequence, in order to include that patient in the study. The use of i2b2 graphical interface proved to be challenging when trying to faithfully reproduce the cohort selection criteria in the data requests with complex temporal criteria, although such criteria can be recreated properly using SQL queries.

Sequences of events could potentially be modeled in the i2b2 hive as a single, pre-coordinated concept, but would require the addition of such concepts to the ontology, as well as pre-processing of the data for identifying such concepts within the observations. Such an approach may still be inadequate in trying to fulfill data request #8, where the researcher had requested a list of patients who had an orthopedic procedure performed, and were still in the therapeutic range for their International Normalized Ratio (INR; therapeutic range: 2.0-3.0) on the day of the surgery. This type of a query would not only require a specific sequence of events to be recorded, but also the interval between these events, or a window of time between successively occurring events, which can not be done using the i2b2 workbench at present. Similar temporal criteria are involved in the case of co-occurring events. The i2b2 workbench does not currently allow for finding such events automatically. In data request #5, for example, the researcher had requested cohorts based on whether the drugs Propofol and/or Lorazepam were administered while the patient was still intubated. The timeline visualization plug-in of the i2b2 workbench allows for visualizing these two events together, and then selecting patients according to the criteria above. This method, however, still relies on manual intervention; the i2b2 workbench does not allow for such criteria to be specified directly, which could otherwise be accomplished using SQL.

Some of the challenges related to modeling, storing and retrieving temporal data from a clinical information system arise from the limitations in how these systems are used to capture the information during clinical care. E.g. diagnosis codes for billing are often entered by specialized medical coders after the clinical encounter has been completed on the basis of information that was captured in the clinical chart in a structured or narrative form. In order to determine an exact point in time when a certain disease *begun*, and when it was *diagnosed*, it would be important to note the information in the electronic chart in a structured, coded manner. Diagnosis related information is often buried in textual narratives, which makes it less accessible to automated retrieval methods without sophisticated methods for processing text, assuming that the exact 'starting point' information was captured at all. The end-point of this initial observation of when a given disease or condition was *resolved *is even harder, since such temporal information is captured less often than when the diagnosis was first made. The limitations that exist in the capture of such clinical information apply equally when trying to retrieve it from the clinical system. E.g. trying to query other clinical conditions or medications or lab results that preceded or followed a given diagnosis from the electronic chart is limited by how effectively the diagnosis information was captured and stored in the chart during the patient's care. Apart from diagnoses, other clinical data such as surgical procedures, lab results, medication administration, etc. already contain the necessary temporal data elements needed to faithfully reproduce the sequence of their occurrence; however, combining them with diagnoses would require some degree of pre-processing in order to allow for queries with temporal criteria, even when directly using SQL as the method for data-retrieval.

### Calculated or aggregate values

About 33% (9) of the data requests contained some inclusion/exclusion criteria consisting of calculated fields or aggregate data, both of which could not be performed using the current version of the i2b2 workbench. In data-request #11, for example, the researcher had requested a cohort of patients where the nadir and peak values for platelet counts were below or above a certain threshold, or where the average value for INR was above the therapeutic range. In the absence of a way for querying based on aggregate functions, this problem could be addressed by post-processing the observations after obtaining an initial data set, although increasing the amount of post-processing would essentially negate the benefits of having an interactive cohort-selection software tool. Version 1.3 of the i2b2 hive software improves the ability to include conditions based on the values of certain findings. This functionality is beneficial in various other types of queries, but it does not directly address the needs of aggregate inclusion/exclusion criteria such as those found in data request #11. On the other hand, the plug-in architecture of i2b2 lends itself well to having more functionality being added in future releases.

### Institution-specific data requests

About 26% (7) of the data-requests that could not be performed using the stock version of i2b2 also contained one or more institution-specific criteria. The use of such criteria in the queries would require a significant amount of pre-processing in the form of data integration from disparate systems and transformation before these could be used with the hive software. As an example, in data request #21, the researcher had requested an estimate of cohort size based on criteria that included data from a system used in the non-invasive Cardiology lab, which is a homegrown ancillary system not currently integrated with the rest of the clinical data warehouse. In spite of not being well integrated, it is still possible to query data from this system using the database backend from within the clinical data warehouse, although making these data available within the i2b2 hive would require developing a regular Extract Transform Load (ETL) process for this system, along with integrating the dictionary for this application within the i2b2 metadata. Conceptually, importing data from the Cardiology system into the i2b2 repository would be similar to integrating these data within the existing clinical data warehouse, although integrating with our existing data warehouse allows for greater flexibility, since it does not limit us to using the i2b2 data model.

Another issue encountered during the analysis was related to data requests that relied on a unique local resource that is independent of the EDW: the Utah Population Database (UPDB) [31-33], which contains family and pedigree information for a large number of people in the state of Utah. Due to the sensitive nature of the data stored in the UPDB and policies governing the use of these data, it is not permitted to link medical records from the EDW directly to genealogical records within UPDB. The linking of UPDB data with other clinical data in the EDW is accomplished using a combination of record-matching and de- & re-identification methods that are consistent with the governance of the resource. Currently, these methods can not be reproduced directly within an i2b2 hive without requiring substantial modification to the underlying code (potentially developing a new cell). Therefore, data requests that included data from the UPDB or other ancillary systems that have not yet been fully integrated within the EDW could not be fulfilled using i2b2.

### The i2b2 data model

**Figure 2:**
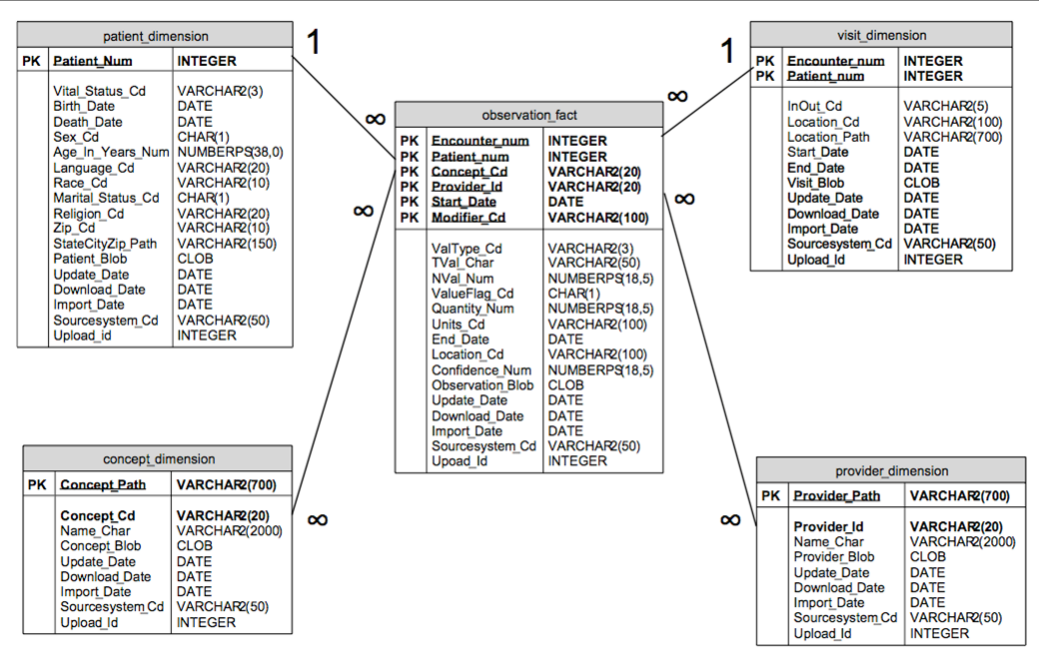
**i2b2 data model**. The i2b2 hive version 1.2 data model star schema showing the core tables (adapted from i2b2 hive documentation [5]).

The i2b2 data model (Figure 2) consists of an Entity Attribute Value Pair (EAVP) derived star-schema, which allows data from sources that have different underlying data models to be transformed and imported into the i2b2 repository (i.e. Clinical Research Chart). However, depending on how the individual contributing systems had been designed, implemented and configured, data in these source systems could be stored in a complex manner, and not all constraints related to referential relationships or embedded/implied logic could be faithfully recreated under the i2b2 model. In particular, trying to reproduce data captured through certain structured clinical documentation posed a unique challenge, since the latter had been created in a variety of different ways across different commercial EMR solutions implemented at our institution.

**Figure 3:**
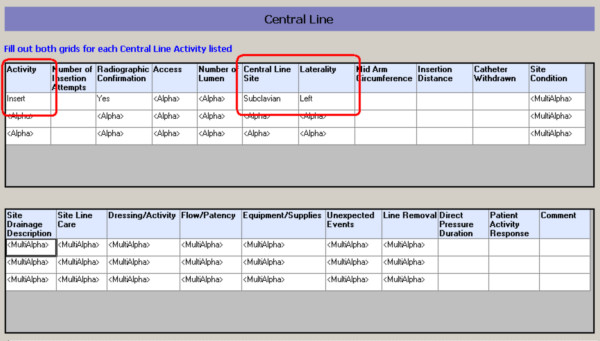
**Structured clinical documentation**. Screenshot of a clinical form for central line activity showing atomic concepts charted separately in a 'grid.' The concepts *central line activity*, *site *and *laterality *are linked within the EMR database, but can not be linked in the i2b2 hive.

An example of some of the problems related to importing data from structured clinical documentation can be found in the clinical form in Figure 3 where individual atomic clinical observations can be charted in a 'grid.' Within the context of the clinical application, the individual observations derive meaning from the column headings, and these meanings can be reproduced within the i2b2 model by creating individual concepts representing 'laterality.' However, the 'type of activity' (first column) can be an insertion, assessment or a removal of the central line, and this information is preserved by linking the concepts within the EMR database. In figure 3, the information that can be charted in a single element within the grid is inherently complex in nature, deriving context from the central line number (row number in the grid), all of the different columns in the grid, the grid itself and the section that contains the grid. In addition, many several individual cells can allow for inputs from multiple-choice popups (labeled 'MultiAlpha'), and a clinical observation charted in this manner can be rich in content & context.

The i2b2 data model has some provision for linking related concepts together within the fact table 'observation_fact,' in the form of a column called 'modifier_cd' which can contain information related to the ranking of modifiers. In order to faithfully reproduce a single data-element described above with applicable contexts, etc., all of these relationships and contexts would have to be expressed within the confines of a single modifier ranking scheme in the 'modifier_cd' column, which can take the form '1,2,3...,' '1.1, 1.2, 1.3, ...,' '1.1.1, 1.1.2, 1.1.3,' according to i2b2 hive documentation. Depending on the complexity of the structured clinical documentation that the source data were charted in, the modifier ranking scheme can quickly prove to be inadequate in being able to faithfully reproduce individual observations that were captured in our EMR system, except by very extensive pre-processing and/or pre-coordinating each of the different possible observations. The 'modifier_cd' column is not currently used by the visual query client tool, which leaves the end-user with no way of querying the data using relationships between observations, even if it were possible to reproduce such relationships within the i2b2 data model. The implications of this limitation are that in the above example, the 'line insertion site' and 'laterality' are charted as separate atomic concepts in the clinical documentation that are linked together in the EMR database. Upon importing the linked observations into the i2b2 repository, the simple ranking/order relationship between them can be preserved at the database level, but the complex spatial relationships between all the different charted fields will be lost. Given the limitations in support for preserving such relationships between observations, this would lead to three separate observations for 'Insert,' 'Left' and 'Subclavian,' with no clinically meaningful means of querying these observations using the i2b2 visual query tool.

The above limitation is an important shortcoming of the current i2b2 data model, and due to this limitation, at the time of writing, individual clinical observations can only be stored and retrieved together at the level of a given 'encounter' or a 'visit' but not at the level of a series of atomic observations that were charted together to convey a clinical fact. The limitation has implications in all post-coordinated clinical concepts which cannot be linked together within the i2b2 data model without creating another pre-coordinated metadata concept to represent these concepts (i.e. different concepts representing every possible combination of activity type, location and laterality). The above limitation, along with the current lack of standardization among EMR vendors may limit the researchers' ability to use data in a consistent manner regardless of their source.

### Updating the i2b2 repository

In order to maintain an up to date research data repository using the i2b2 hive, a shadow copy of the i2b2 tables containing additional data attributes and metadata that allow for tracking changes to clinical observations would be required since it is not possible to completely manage such attributes within the i2b2 star schema itself. E.g. tracking changes to laboratory observations like Microbiology results can be a challenging task when cultures are ordered on a given day, followed by the specimen being collected, preliminary results being reported, and finalized results being reported in 24-72 hours, depending on the type of cultures or antibiotic sensitivity tests ordered. In order to keep track of changes occurring to these results over a period of about 72 hours, and for keeping related results together, there would be a need for a consistent identifier at the level of the specimen or lab test order number, which would require maintaining a list of these separately.

According to the i2b2 documentation, such identifiers can be maintained at the visit level in the 'visit_dimension' table, which represents 'sessions where observations were made' and can contain 'visits, events or encounters.' Although visits & encounters are often used synonymously, using the visit dimension to also store consistent identifiers at the level of specimen or lab test number can lead to situations where observations that were part of a panel of lab tests ordered at the same time can be linked together using these identifiers, but observations from different panels of lab tests could not be grouped together easily. E.g. if two lab panels were ordered during the same inpatient hospital visit, each of the different the panels could now have different lab test order numbers, and if these were to be stored separately in the visit dimension, the individual results from lab panel #1 could be grouped together, as could be the individual results from lab panel #2, but there would be no way of grouping all the lab test results from both of the panels that were obtained during the same hospital visit, since the identifier 'encounter_num' would be different for results from lab panels 1 & 2, thus leaving the user with limited or no ability to search for all related lab test results during a single hospital visit using the visual query tool. A more elegant solution to this problem would be to introduce another dimension in the i2b2 data model which maintained the 'grouping events' (e.g. lab panel order number, or an order-set order number) separately from the true visits or encounters stored in the visit dimension.

The core i2b2 fact table (observation_fact) does not currently have provision for managing such data elements, and therefore, resolving status changes on results or managing updates to clinical observations need to be performed in a 'staging area' outside of the core i2b2 tables, before uploading/updating the data in the repository. A majority of the research data requests in the sample consisted of retrospective review, where the timeliness and currency of data in the i2b2 repository were of secondary importance to the richness of attributes available. Ultimately, any institutional strategy for keeping the i2b2 repository updated would have to take into consideration the use cases for research data requests that would be performed using i2b2.

## Software availability and requirements

The i2b2 hive and workbench software, as well as the complete source code are freely available for download at http://www.i2b2.org/software/index.html under the 'i2b2 open source license.' [5] Installation of the i2b2 hive requires a Linux [22] or Microsoft Windows server or a workstation with Sun Java Development Kit (JDK) [7] version 5.0 (1.5.0.11 or above), Apache Tomcat [17] version 5.5.23 with JDK 1.4 compatibility package, JBoss Application Server [18] 4.2.x GA, Apache Axis2 [19] version 1.1, Apache Ant [34] version 1.6.5 (or above), Oracle 10 g Express Edition [21] (or above) or Microsoft SQL Server. The i2b2 workbench is available for Microsoft Windows and Apple Macintosh platforms. Alternatively, a fully functional virtual machine image of the hive is available for evaluation at the above location, and using this image requires VMWare [35] virtualization software running on a Windows/Mac/Linux host operating systems.

## Conclusion

The availability of structured and coded data, along with rich metadata, are key to ensuring success in any kind of data integration initiative, and some of the issues encountered during populating the repository for the i2b2 hive underscored this lesson. Data from legacy systems present particular problems with regard to integration that span far beyond the i2b2 hive; these problems need to be addressed by the adoption of controlled medical terminologies, mapping legacy data and generating rich metadata allowing their reinterpretation in the context of newer discoveries. The i2b2 data model presented some challenges with regard to the type of data requests that could be fulfilled using the i2b2 workbench for selecting patient cohorts in clinical studies. Some of these challenges were not unique to this software tool, but could also present themselves when using commercially available products that are based on similar, underlying data models. The inadequacy of provisions for preserving contexts and relationships between individual clinical observations within the i2b2 repository is a significant shortcoming of the model. This shortcoming is especially important when importing data from structured clinical documentation into the i2b2 hive, since a consequence of this limitation is that every atomic observation will have to be available as a pre-coordinated concept, which is not practical in an environment with a large-scale EMR implementation. In conclusion, the i2b2 hive could potentially be a valuable tool for clinical research, provided that structured and coded clinical data and metadata are available, which can be transformed to fit the i2b2 logical data model, and sound strategies are adopted for pre- and post-processing these data based on relevant use cases from a given institution. An institution that decides to use the i2b2 hive could expect to have a considerable volume of simple queries off-loaded from data analysts into the hands of researchers using the i2b2 hive directly, freeing up the analysts to focus their attention on the more complex and challenging problems.

## Competing interests

VGD and SMM received partial salary support from the Harvard i2b2 subcontract award (NCBC U54 LM008748), at the University of Utah, during the period 2007-2008.

## Authors' contributions

As a senior faculty mentor, JAM conceived the study; VGD planned the architecture, installed the hive and analyzed the electronic data requests according to different criteria along with SMM. All of the authors participated in the study design, coordination, and in preparing the written manuscript.

## Pre-publication history

The pre-publication history for this paper can be accessed here:

http://www.biomedcentral.com/1471-2288/9/70/prepub

## Supplementary Material

Additional file 1 **'Description of the data requests presented in tables **1 &2'. 'Additional file 1' contains descriptions of the data requests as they were entered in the data-request tool, and the numbering is consistent with that used in tables 1 &2 in the manuscript. Click here for file
