# Supplementary material for: Evaluating the informatics for integrating biology and the bedside system for clinical research
Source: BMC Med Res Methodol. 2009 Oct 28;9:70. doi: 10.1186/1471-2288-9-70 (PMC2779809; doi:10.1186/1471-2288-9-70)
Supplement: Additional file 1 — 'Description of the data requests presented in tables 1 &2'. 'Additional file 1' contains descriptions of the data requests as they were entered in the data-request tool, and the numbering is consistent with that used in tables 1 &2 in the manuscript. [file 1471-2288-9-70-S1.doc]

**Appendix I:** Description of the data requests presented in Tables 1 & 2

| **Req #** | **Description** |
| --- | --- |
| 1 | PI is looking at fungal infections in MICU patients, and needs to know what antibiotics patients received and for what periods of time. Specifically interested in looking at administration of IV antibiotics in this cohort while the patients were intubated. |
| 2 | PI needs counts of patients that had Basal cell Carcinomas, Squamous Cell Carcinomas, Squamous Cell Carcinomas in situ, Displastic Nevus, any kind of Nevus, melanomas, etc. Broken down by years 2004 and 2005 |
| 3 | PI would like to look at all stroke patients, and would like the length of stay with and without outliers (outlier = 3 x std dev). |
| 4 | PI would like a list of patients who had hip/knee surgery and had received Rifampicin after their surgery. |
| 5 | PI is interested in looking at patients who were intubated greater than 48 hrs and received Propofol or Ativan while being intubated. |
| 6 | Female patients from January 2002 onwards with a diagnosis of DVT/PE and age less than 51. |
| 7 | PI would like trending data for her clinic INR(International normalized ratio) with the number of monthly INRs, number of critical INRs, percentage of INR within therapeutic range, diagnoses & total number of patients in the clinic. |
| 8 | PI needs a list of patients who have undergone Orthopedic surgery with a therapeutic INR on the day of surgery. |
| 9 | PI needs to find d-dimer values, dates & times on patients who have undergone Neurosurgery. |
| 10 | PI has requested list of names, MRNs of patients receiving Methylprednisolone gtt for acute spinal cord injury, including dates and doses for the period 01/01/2001 to 01/01/2007 |
| 11 | PI has requested data on patients with TTP, or who have undergone plasmapheresis, or who have had the test ADAMTS13 ordered on them. The data elements requested contain CBCs, Chemistry panels, PT/INR and other labs with nadir and peak values for each of the lab results. |
| 12 | List of patients who have had D-dimer tests ordered or duplex ultrasound studies performed, along with the test/procedure dates and test-results. |
| 13 | PI needs data on patients with a diagnosis of Hypertension or systolic blood pressure greater than 130 mm Hg. Data elements include comorbidities (Diabetes, CAD, Stroke, etc.), procedures (CABG, coronary angiography, etc.) age and other patient demographics. |
| 14 | PI wants a report of all admits for patients with asthma, COPD, emphysema, atopic dermatitis or hay fever. |
| 15 | A list of pediatric burn patients (defined as age less than 15 years, and ICD-9 diagnosis codes 940-949, including all the sub-codes), who received low-molecular weight Heparin (Enoxaparin) during their hospital-stay, and who had anti-Factor Xa levels measured. |
| 16 | This study will evaluate the use of Echocardiography in driving management decisions in critically ill burn patients. PI needs to identify all burn patients (ICD Codes 940-949) during the last 5 years who had an Echo procedure. |
| 17 | This is a pre-research data request for an estimate of sample size on patients with non-ruptured cerebral aneurysm (ICD Code 437.30) who have had at least two Head MRA procedures. |
| 18 | PI needs a list of all ICU patients with a separate list for SICU patients who had an admission weight greater than 120kg. |
| 19 | List of patients with STEMI / PCI from 4/1/2008 - 6/30/2008 |
| 20 | Need total number of new patients with the following diagnosis (ICD-9) codes billed from January - June 2008 by three physicians. Diagnosis codes: 596.59, 596.51, 788.31, 788.63, 788.41, either as primary or secondary diagnoses. |
| 21 | PI needs an estimate of how many patients were diagnosed with Aortic Stenosis from the Cardiology ‘Lynx’ application database that is used in the non-invasive cardiology lab. |
| 22 | Number of cases of childhood epilepsy and how many of these link to third generation families in the UPDB. Since multiple cases occur in a single family, user has requested both datasets: number of cases from the data warehouse as well as from the UPDB. |
| 23 | User needs a count from the Enterprise Data Warehouse of the patients with stroke. If possible, she would like to also know how many of them link to the UPDB. |
| 24 | The number of interventional cases (any case with PTCA, stent, alcohol ablation, PFO closure, ASD closure). List the number of cases by year from 2006, 2007, and 2008 |
| 25 | PI has requested the following data-elements: Main hospital MRNs, patient names, discharge dates, times, units, discharging physician, discharge disposition, discharged with prescriptions; Patient-care units: 6N |
| 26 | PI has requested the number of CABG cases alone or with other types of procedure per month since June 2006 to the present. |
| 27 | Patient level detail for all patients that have had cardiac surgery since 7/1/2008 |
